# Structure–function relationship in nano ZnO-curcumin reinforced pea protein films prepared via high-pressure homogenization

**DOI:** 10.1016/j.fochx.2026.103824

**Published:** 2026-04-11

**Authors:** Gulsah Karabulut, Vedant Mundada, Ragya Kapoor, Hao Feng

**Affiliations:** a Department of Food Engineering, Faculty of Engineering, Sakarya University, 54187, Sakarya, Türkiye; b Department of Food Science and Human Nutrition, University of Illinois at Urbana-Champaign, 61801 Urbana, IL, USA; c Urban and Community Food Complex, College of Agriculture and Environmental Sciences, North Carolina A&T State University, Greensboro, NC 27401, USA

**Keywords:** Zinc oxide nanoparticles, Curcumin, Bioactive films, Food simulants, Sustainable packaging

## Abstract

The design of sustainable, active packaging materials offers a promising alternative to petroleum-based plastics. This study investigated the integration of zinc oxide nanoparticles (ZnO NPs) and curcumin (Cur) into pea protein isolate (PPI) films using high-pressure homogenization (HPH) to enhance structural and functional performance. ZnO NPs were synthesized via ultrasound-assisted precipitation, and curcumin was added at 0.075–0.375%. HPH treatment promoted uniform dispersion and improved film cohesion. HPH-treated films exhibited enhanced crystallinity, mechanical strength (tensile strength up to 1.94 MPa), and flexibility (up to 50.97%). Importantly, HPH reduced water vapor permeability (WVP), and the Cur–nZnO films showed the lowest WVP (3.9 ± 0.3 × 10^−10^ g·m/(m^2^·s·Pa)) compared with HPH-treated and control films, indicating improved moisture barrier performance. Thermal stability improved with a higher degradation onset and residual mass due to the thermal resistance of ZnO. Optical analysis revealed that Cur–ZnO NPs films had the lowest UV–Vis transmittance, supporting their UV-blocking capability. Bioactivity assessments confirmed that Cur–ZnO NPs films achieved 1.3–1.6 log CFU/mL reductions in bacterial counts against *Listeria innocua* and *Escherichia coli*. Surface analysis via XPS confirmed successful integration of Cur and ZnO, with enrichment in oxygenated and zinc-related groups. Release studies in ethanol, acetic acid, and oleic acid revealed simulant-dependent kinetics. Cur followed the Peleg model behavior, with the highest release in acetic acid. Zn^2+^ release fit a zero-order model in ethanol, and diffusion was faster in acid. These results demonstrate the potential of HPH-processed Cur–ZnO NPs PPI films for active packaging, offering tailored barrier and antimicrobial functions.

## Highlights

• ZnO nanoparticles and curcumin were incorporated into pea protein films via HPH.
• HPH enhanced film crystallinity, tensile strength, and flexibility.
• Cur–nZnO films exhibited improved UV-blocking, thermal, and antimicrobial properties.
• XPS and FTIR confirmed effective ZnO–curcumin–protein interactions.
• Curcumin and Zn^2+^ release followed kinetic models, with the fastest diffusion in acetic acid.

## Graphical abstract

**Figure:**
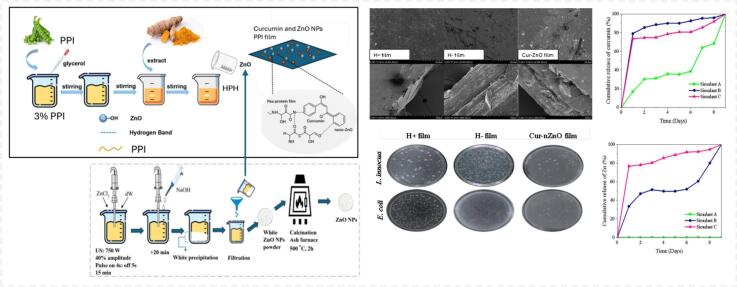

## 1 Introduction

Increasing awareness of the environmental consequences of conventional plastic packaging has driven growing interest in sustainable materials for single-use food applications. (Wei et al., 2023). Conventional plastics, while offering excellent barrier and mechanical properties, are non-biodegradable and contribute significantly to microplastic pollution and greenhouse gas emissions throughout their life cycles (Bonilla et al., 2012). In this context, bio-based alternatives, especially edible films derived from renewable, biodegradable sources, have emerged as promising solutions to address the ecological drawbacks of plastic packaging (Tang et al., 2019a, 2019b).

Among various biopolymers, plant-derived proteins, such as pea protein isolate (PPI) stand out for their biodegradability, renewability, and nutritional attributes (Ge et al., 2020). PPI, a legume-based protein, is abundant in globulins (legumin and vicilin) and exhibits good film-forming capability due to its amphiphilic amino acid composition (Luo et al., 2022). However, native pea protein films suffered from structural and functional limitations, including low tensile strength and poor antimicrobial performance (Cheng & Cui, 2021). These shortcomings stem from the globular nature of pea proteins and their limited interchain interaction in the dry state, which results in heterogeneous, poorly ordered film networks (Lan et al., 2018).

To overcome these limitations, the incorporation of functional nanomaterials and bioactive compounds into protein-based films has been widely explored. Zinc oxide nanoparticles (ZnO NPs) are among the most extensively studied inorganic nanofillers for food packaging applications due to their broad-spectrum antimicrobial activity, UV-shielding capability, and mechanical reinforcement potential (Channa et al., 2022; Roy & Rhim, 2020a). ZnO NPs exert their antimicrobial action by interacting with bacterial cell membranes, generating reactive oxygen species (ROS), and releasing Zn^2+^ ions that disrupt cellular metabolism (Huang et al., 2021). Moreover, due to their nanoscale dimensions, ZnO NPs can occupy the interstitial spaces in biopolymer matrices, decreasing porosity and improving barrier properties (Tang et al., 2019a, 2019b).

Previous studies have demonstrated that incorporating ZnO NPs into soy protein, whey protein, or cellulose-based films significantly improves their antimicrobial efficiency and UV-barrier performance, while enhancing tensile strength and decreasing water solubility (El Fawal et al., 2020; Ran et al., 2022; Shi et al., 2008). Furthermore, the combination of ZnO with polyphenolic additives such as mangosteen peel extract or anthocyanins has shown synergistic effects in biopolymer matrices (Huang et al., 2021; Ran et al., 2022). However, many of these studies rely primarily on formulation-based strategies and conventional dispersion methods, often overlooking how processing-induced structural reorganization governs film performance and active compound release.

Curcumin, a lipophilic polyphenol derived from *Curcuma longa*, adds further functionality as a natural antioxidant, antimicrobial, and anti-inflammatory agent (Aliabbasi et al., 2021). However, its high crystallinity, poor water solubility, and susceptibility to photodegradation hinder its bioavailability and incorporation in aqueous biopolymer systems (Wang et al., 2022). As a result, achieving uniform curcumin distribution and controlled release within protein-based films remains a major challenge.

Physical modification techniques have therefore been employed to improve the dispersion and compatibility of hydrophobic bioactives in protein matrices. Among these, high-pressure homogenization (HPH) has emerged as a scalable and environmentally friendly process capable of simultaneously modifying protein structure and dispersing nano-scale additives (Luo et al., 2022; Song et al., 2013). HPH imposes intense shear, turbulence, and elongational flow fields throughout the entire fluid volume. These forces promote reproducible protein unfolding, enhanced molecular alignment, and stronger intermolecular interactions at the macromolecular scale (Karabulut et al., 2024). Consequently, HPH-treated systems often exhibit improved matrix cohesion, filler–polymer interfacial compatibility, and structural homogeneity (Dong et al., 2011).

In this context, the co-incorporation of ZnO NPs and curcumin into a high-pressure homogenized PPI matrix represents a process-driven strategy rather than a purely compositional one. ZnO NPs primarily contribute to mechanical reinforcement, UV shielding, and antimicrobial protection, while curcumin provides antioxidant activity and pH-responsive functionality. Importantly, HPH-induced protein unfolding and network reorganization may suppress curcumin crystallization, improve nanoparticle dispersion, and generate dense film architectures that enable tunable, simulant-dependent release behavior of both Zn^2+^ ions and curcumin. Such structure–function–release relationships have not been systematically elucidated in previous protein-based active packaging studies.

The novelty of this work lies in elucidating how HPH-mediated protein structuring governs the synergistic performance of dual active agents within a pea protein film matrix. Rather than emphasizing additive effects alone, we propose a mechanistic framework linking processing conditions to microstructure, functional performance, and release kinetics. Specifically, pea protein-based films incorporating ZnO nanoparticles and curcumin were produced via high-pressure homogenization and systematically characterized for physicochemical, mechanical, microstructural, and functional properties (XRD, SEM, FTIR, mechanical testing, and antimicrobial performance). In addition, the release of curcumin and Zn^2+^ was quantified and kinetically modeled in relevant food simulants (10% ethanol, 3% acetic acid, and oleic acid) to elucidate migration mechanisms. Overall, by integrating nanomaterial engineering, protein structuring, and kinetic modeling, this study provides design insights for next-generation multifunctional edible films for active food packaging applications.

## 2 Material and methods

Pea protein isolate (PPI, 85% protein) was donated by AGT Food and Ingredients (Regina, Saskatchewan, Canada). All other chemicals were purchased from Sigma-Aldrich (St. Louis, MO, USA) at analytical or higher purity. *Listeria innocua* (M7M446 B11) and *Escherichia coli* (K12 LB M7M025) bacterial cultures were obtained from the culture collection of Dr. Miller at the University of Illinois at Urbana-Champaign (USA) in the Department of Food Science and Human Nutrition.

### 2.1 Curcumin extract

Curcumin solutions were prepared by dissolving curcumin (0.075%, 0.225%, and 0.375% relative to the total mass of the film-forming solutions) in 2.0 mL of an ethanol-water mixture (ethanol:water = 4:1, v/v). The mixture was sonicated using a 13 mm ultrasonic (US) probe (Sonics, VCX 750, USA) at 450 W for 10 min to ensure complete dissolution (Dong et al., 2023). The resulting curcumin solution was then added to the film-forming solution under continuous stirring.

#### 2.1.1 Synthesis of ZnO NPs

**Fig. 1:**
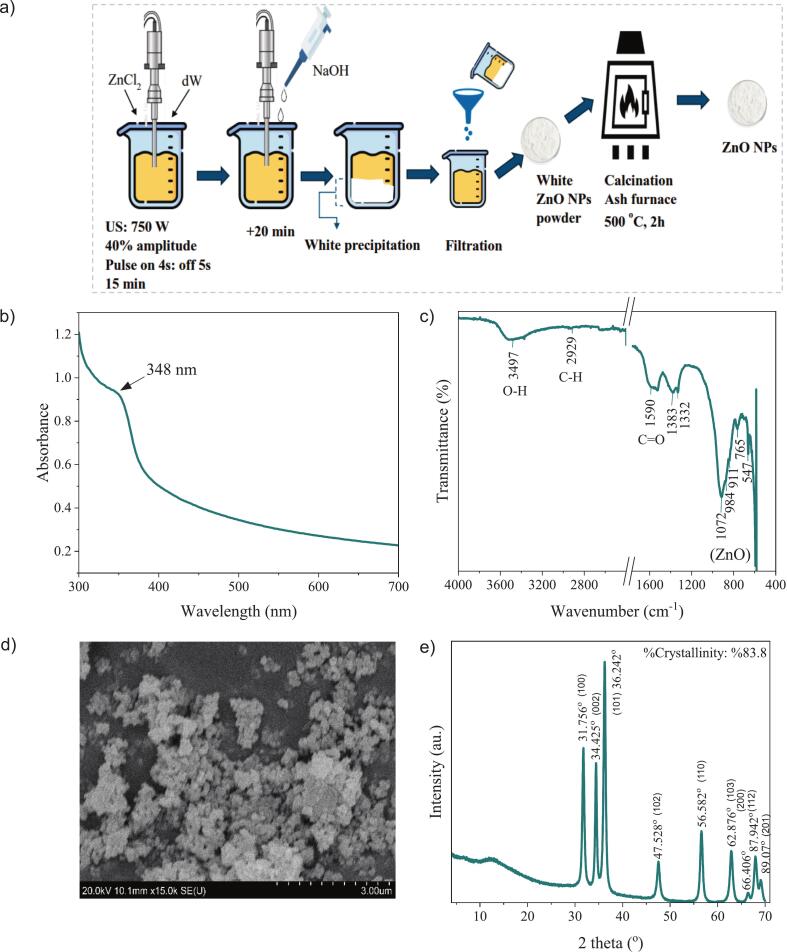
a) Synthesis and characterization of ZnO NPs using b) Spectral scan, c) FTIR, d) SEM images, and e) XRD spectra.

The modified method was employed to synthesize ZnO NPs (Bhanvase et al., 2019) (Fig. 1a). A zinc chloride (ZnCl₂) solution was prepared by dissolving 2.2 g of ZnCl₂ in 12 mL of distilled water (DW), followed by ultrasound (US) treatment (Sonics, VCX 750, USA) at 450 W (4 s: 5 s on: off) for 15 min. A 2 N sodium hydroxide (NaOH) solution (50 mL) was added dropwise at 10 mL/min under continuous sonication to precipitate ZnCl₂ and initiate ZnO NPs formation. Sonication continued for an additional 20 min under the same conditions. After nucleation, the reaction mixture was filtered using coarse filter paper, and the precipitate was washed with DW until a neutral pH was achieved. The product was calcined at 500 for 2 h in an ash furnace to crystallize ZnO NPs, then manually ground into a fine powder. The resulting ZnO NPs were characterized using spectral scanning, FTIR, SEM, and XRD techniques as detailed below.

#### 2.1.2 Spectral scan of ZnO NPs

To perform a spectral scan using UV–Vis spectroscopy (Thermo, Spectronic Genesys 5, USA), a colloidal solution was prepared by dispersing 0.1 g of ZnO NPs in 10 mL of ethanol. The solution was homogenized using ultrasonication for 10–15 min to ensure uniform dispersion. The UV–Vis spectrophotometer was calibrated with ethanol as the blank. The ZnO NPs solution was then transferred to a clean quartz cuvette, ensuring it was free of air bubbles, and the absorption spectrum was scanned over 300–800 nm (Bhanvase et al., 2019).

### 2.2 Preparation and characterization of ZnO NPs/Curcumin-loaded pea protein films

**Fig. 2:**
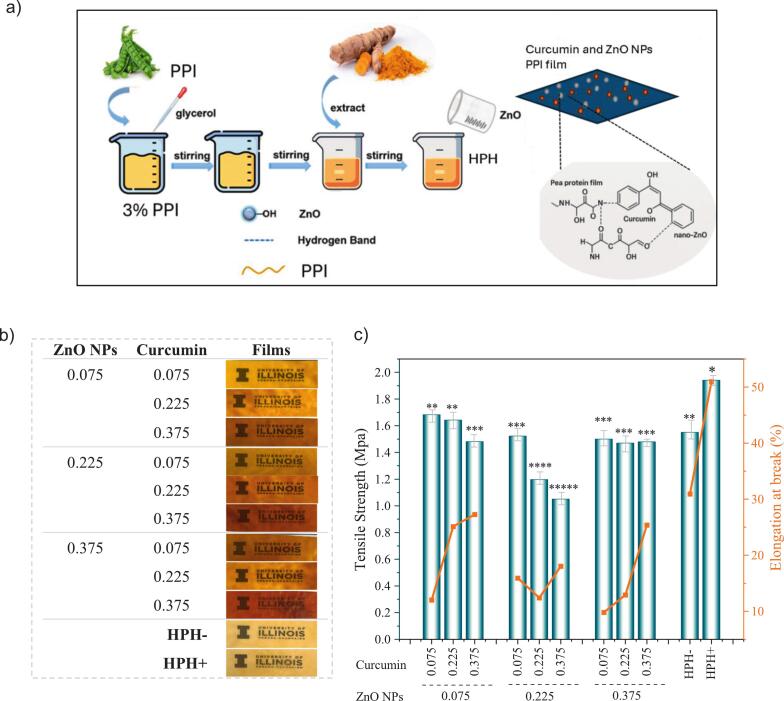
a) Scheme of the preparation of curcumin and ZnO NPs containing pea protein films, b) visual of the different ratios of curcumin and ZnO NPs incorporated into films, c) mechanical properties of pea protein films with/withouh high pressure homogenization and curcumin and ZnO NPs.

The resulting film solutions were processed using a high-pressure homogenizer (Microfluidics M110P, USA) at 130 MPa for three passes, corresponding to an effective homogenization time of approximately 3–4 min, to ensure uniform dispersion and protein structural modification (Fig. 2a). Control samples consisted of homogenized (HPH^+^) and non-homogenized (HPH^−^) film-forming solutions without ZnO nanoparticles and curcumin.

Following homogenization, the film-forming solutions (20 mL) were degvassed under vacuum for 20 min to remove entrapped air bubbles, cast onto square polytetrafluoroethylene dishes (9 × 9 cm^2^), and dried at 50 °C for 24 h. The dried films were then conditioned at 25 °C and 50% relative humidity (RH) for 48 h prior to characterization. Homogenized (HPH^+^) and non-homogenized (HPH^−^) protein-only films were prepared as process controls to evaluate the effect of high-pressure homogenization on pea protein structuring and film formation. Composite films containing ZnO NPs and curcumin were prepared exclusively under HPH conditions to ensure uniform dispersion and reproducible film quality. Preliminary non-homogenized composite formulations resulted in pronounced nanoparticle agglomeration and poor film integrity, and were therefore excluded from systematic characterization.

#### 2.2.1 Mechanical properties

The mechanical properties of films (dimensions: 15 mm × 80 mm) were evaluated using a texture analyzer (Brookfield, Ametek CT3, Middleboro, MA, USA) equipped with a TA-DGA tensile grip and a 0.4 N load cell in accordance with ASTM D882. Measurements were performed at a crosshead speed of 0.40 mm/s with an initial grip separation of 50 mm. Tensile strength (TS) and elongation at break (EAB, %) were calculated using the texture analyzer software (Brookfield, USA).

#### 2.2.2 Fourier transform infrared spectroscopy with attenuated total reflectance (FTIR-ATR)

Spectra of films and ZnO NPs were taken in the spectral range of 400–4000 cm^−1^ using an ATR-FTIR spectrometer (PerkinElmer Spectrum Two, USA) at ambient conditions. For each spectrum, 16 scans at a resolution of 4 cm^−1^ were obtained. The films were directly placed onto the diamond ATR crystal without further preparation. The wavenumbers of the Amide I, Amide II, and Amide III regions were evaluated from the graphs.

#### 2.2.3 X-ray diffraction (XRD)

The crystallographic structures of the film samples were analyzed using an X-ray diffractometer (Bruker D8 Advance, Germany) equipped with a Cu-Kα radiation source (λ = 1.5406 Å), operating at an accelerating voltage of 40 kV and a current of 30 mA. The diffraction patterns were recorded over a 2θ range of 5° to 70°, with a step size of 0.02° and a scan rate of 2°/min under ambient conditions, following the protocol described by Demirkesen et al. (2014). The resulting diffractograms were used to calculate the degree of crystallinity using the Diffrac. Eva software (version V5.1, Bruker, Rheinstetten, Germany).

#### 2.2.4 Scanning electron microscopy (SEM)

The morphology of the films and ZnO NPs was examined using a scanning electron microscope (S-4700 SEM, Hitachi, Ltd., Tokyo, Japan). Prior to imaging, samples were mounted on aluminum stubs using double-sided carbon tape and sputter-coated with a thin layer of gold (approximately 10 nm) using a sputter coater under vacuum for 30 s to enhance conductivity and prevent charging. The top (air-exposed) and cross-sectional surfaces of the samples, fractured in liquid nitrogen to prevent deformation, were examined under high vacuum at 5 kV using SEM at magnifications of ×15,000, ×2000, and ×500.

#### 2.2.5 Thermogravimetric analysis (TGA)

The thermal stability of the films was evaluated using a thermogravimetric analyzer (TA Instruments Q2500, New Castle, USA). Approximately 50 mg of each sample was heated from room temperature to 600 °C at a rate of 10 °C/min under a nitrogen flow of 40 mL/min. A platinum crucible was used, with α-Al₂O₃ as the reference material (Azevedo et al., 2020). Thermogravimetric (TG) and derivative thermogravimetric (DTG) curves were simultaneously recorded to identify weight loss stages.

#### 2.2.6 X-ray photoelectron spectroscopy (XPS)

Surface elemental composition and chemical states were analyzed using a XPS system (Kratos Analytical Ltd., UK) with a monochromatic Al Kα source (1486.6 eV, 15 kV, 150 W). Survey spectra (0–1100 eV) and high resolution spectra (e.g., C1s, O1s, N1s, Zn2p) were recorded at pass energies of 160 and 20 eV, respectively. Charge correction was applied using the C1s peak at 284.8 eV. Data analysis and peak fitting were performed with CasaXPS software (Huang et al., 2021; Li, Long, et al., 2024; Li, Wei, et al., 2024).

#### 2.2.7 Transmittance of films

The optical transmittance of the films was determined using a UV–visible spectrophotometer (Thermo, Spectronic Genesys 5, USA). equipped with a quartz cuvette. Film samples were carefully cut into uniform rectangular strips and placed directly in the cuvette holder without folding or overlapping. The transmittance (%) was recorded in the wavelength range of 300–800 nm at 1 nm intervals under ambient laboratory conditions. Air (empty cuvette) was used as the baseline reference.

#### 2.2.8 Water vapor permeability (WVP)

Water vapor permeability (WVP) was determined according to Karabulut et al. (2025), with minor modifications. Briefly, 5.0 g of anhydrous calcium chloride (CaCl₂) was placed into permeation cups, and film specimens were tightly sealed over the cup openings to prevent edge leakage. The cups were weighed to obtain the initial mass and then placed in a controlled chamber at 85 % relative humidity and 37 °C. The mass increase, attributed to water vapor transmission through the film, was recorded at predetermined intervals until a steady state was reached. WVP was calculated using Eq. (1):

(1) $$\mathrm{WVP}=\frac{w.d}{A_{t}.\mathrm{\Delta P}}$$

where w is the moisture gain (g), d is the film thickness (m), A is the exposed film area (m^2^), t is the exposure time (s), and ΔP is the water vapor partial pressure difference across the film (Pa).

#### 2.2.9 Antibacterial activity

The antibacterial activity of the films was evaluated against Gram (+) *Listeria innocua* M7M446 B11 and Gram (−) *Escherichia coli* K12 LB M7M025. Sterile film samples (cut into discs or 2 × 2 cm pieces) were aseptically added to 10 mL of the bacterial suspensions (9 log CFU/mL) in sterile tubes. During incubation at 37 °C, aliquots were taken at 0, 2, 24, and 48 h. At each time point, 100 μL of appropriate dilutions were surface-plated on Plate Count Agar (PCA). After incubation at 37 °C for 24 h, colonies were counted to determine the viable cell count (CFU/mL). The antibacterial effect was evaluated by comparing the bacterial counts over time with a control group (bacterial suspension without film) (Amankwaah et al., 2020).

#### 2.2.10 Release of curcumin and ZnO NPs into food simulants

According to modified method of Cano et al. (2016), three food simulants were used: A (10% ethanol), B (3% acetic acid), and C (oleic acid). Film samples (6 cm^2^) were immersed in 20 mL of simulant and incubated at 25 °C for 9 days. At set intervals, simulant aliquots were diluted (with water for A and B; ethanol for C) and analyzed. Zn release was quantified via ICP-OES (PerkinElmer, Optima 8300, USA) using Zn calibration curves and expressed as % Zn release. For curcumin release, 10 mg of the loaded sample was dispersed in 10 mL methanol, stirred in the dark for 3 h, and centrifuged (9000 ×*g*, 20 min). The absorbance of supernatant at 425 nm was measured using UV–vis spectroscopy, and % Curcumin release was calculated based on a standard curve.

### 2.3 Statistical analysis

All analyses were carried out in three replicates and presented as mean ± standard deviation. Statistical analyses were done using an SPSS 20.0 package program (SPSS Inc., Chicago, USA). The results were tested with a one-way ANOVA. A *p*-value was applied at a 95% confidence interval.

## 3 Results and discussions

### 3.1 Characterization of ZnO NPs

The synthesized ZnO NPs were characterized using UV–visible spectroscopy, FTIR, SEM, and XRD analyses to confirm their formation, structure, and morphology (Fig. 1a–e). Although several studies have reported the synthesis of ZnO NPs from waste-derived precursors, such approaches often suffer from compositional heterogeneity and limited control over particle purity and surface chemistry. In the present study, a controlled ultrasound-assisted precipitation route was deliberately selected to obtain structurally well-defined and reproducible ZnO NPs, enabling reliable evaluation of their role within protein-based composite films.

UV–vis spectroscopy (Fig. 1b) revealed a strong absorption peak between 340 and 380 nm, which is characteristic of ZnO NPs due to their wide bandgap (∼3.3 eV), consistent with the excitonic absorption of ZnO NPs reported in previous studies (Ran et al., 2022; Shi et al., 2008; Sirelkhatim et al., 2015).

FTIR analysis (Fig. 1c) confirmed the presence of Zn—O bonds with a prominent absorption band observed around 450–500 cm^−1^, which is attributed to the stretching vibrations of Zn—O in the wurtzite lattice. Additionally, broad bands in the 3200–3500 cm^−1^ region correspond to O—H stretching vibrations, indicating the presence of surface hydroxyl groups, possibly from adsorbed water or residual synthesis components, as previously observed by Espitia et al. (2014) and El Fawal et al. (2020).

SEM imaging (Fig. 1d) showed that the ZnO NPs were agglomerated and had irregular yet generally spherical morphology. The particles formed dense clusters, which is commonly attributed to the high surface energy of ZnO NPs, leading to aggregation during drying (Huang et al., 2021; Shi et al., 2008). Despite this tendency, the controlled synthesis ensured consistent particle morphology and size distribution, which is essential for reproducible incorporation into composite films and meaningful structure–property interpretation.

XRD analysis (Fig. 1e) confirmed the crystalline nature and phase purity of the synthesized ZnO NPs. The diffraction peaks were in good agreement with the standard hexagonal wurtzite structure of ZnO (JCPDS Card No. 36-1451), showing characteristic reflections at 2θ ≈ 31.7° (100), 34.4° (002), 36.2° (101), 47.5° (102), 56.6° (110), 62.8° (103), and 68.0° (112), consistent with prior reports (Madian et al., 2023; Shi et al., 2008). The presence of intense and sharp peaks, particularly at (101), (002), and (100) planes, indicates high crystallinity (∼83.8%). Importantly, the absence of secondary or impurity-related peaks confirms that the adopted synthesis route yields phase-pure ZnO NPs, which is critical for isolating the effects of ZnO on film mechanical, antimicrobial, and release properties without interference from uncontrolled by-products (Akshaykranth et al., 2023; Channa et al., 2022).

### 3.2 Mechanical properties

Fig. 2a and b illustrate the preparation scheme and visual appearance of the films, respectively, while the effects of varying ZnO NPs and curcumin concentrations on TS and EAB are shown in Fig. 2c.

At the lowest ZnO NPs concentration (0.075%), increasing curcumin from 0.075% to 0.375% resulted in a reduction in TS (from 1.68 to 1.48 MPa), while EAB increased from 12.03% to 27.29%. This suggests that curcumin acts as a plasticizer-like additive at low ZnO levels, disrupting polymer–polymer interactions and increasing chain mobility, thereby enhancing film stretchability (Rachtanapun et al., 2021; Roy & Rhim, 2020b; Wang et al., 2022).

At a moderate ZnO NPs level (0.225%), both TS and EAB exhibited a downward trend with increasing curcumin. This could be attributed to poor interfacial compatibility or agglomeration of ZnO NPs in the presence of excessive curcumin, leading to structural discontinuities and reduced stress transfer between phases (El Fawal et al., 2020; Madian et al., 2023). Interestingly, at the highest ZnO level (0.375%), TS remained relatively stable (1.47–1.50 MPa), but EAB showed a significant rise with increasing curcumin, reaching 25.40% at 0.375% curcumin. This behavior indicates a potential synergistic interaction at higher filler and curcumin loadings, where curcumin may reduce brittleness and improve matrix-filler adhesion, thereby offsetting the rigidity imparted by the high ZnO content (Huang et al., 2021).

Based on mechanical data, the 0.075% ZnO NPs + 0.225% Curcumin formulation was selected as the optimal combination, offering a good balance of strength and flexibility suitable for handling and end-use functionality.

The control samples, non-homogenized (HPH^−^) and homogenized (HPH^+^) films, provided a baseline for assessing the effect of processing on mechanical performance. The HPH^+^ film exhibited the highest TS (1.94 MPa) and EAB (50.97%), demonstrating that high-pressure homogenization substantially improves mechanical integrity under highly plasticized conditions. This enhancement is attributed to HPH-induced protein unfolding, improved molecular alignment, and stronger intermolecular interactions, which promote the formation of a denser, and more cohesive film network (Cheng et al., 2022).

It should be noted that a non-homogenized composite control containing ZnO nanoparticles and curcumin was not included in the present study. This choice was made to prioritize film homogeneity and reproducibility, as non-HPH composite systems exhibited severe agglomeration and casting instability in preliminary trials. Future studies may incorporate such controls to further decouple the individual contributions of processing and formulation variables.

Although the tensile strength values obtained in this study are lower than those of commercial petroleum-based plastics, such comparisons are not directly applicable, as the present films are designed as biodegradable, edible, and bioactive materials rather than structural packaging layers. The relatively high glycerol content (25% of total solids), intentionally used to ensure flexibility, processability, and film integrity, inherently limits tensile strength while markedly enhancing extensibility. Similar mechanical ranges have been widely reported for pea protein and other globular protein-based films formulated with comparable plasticizer levels. From an application perspective, the developed films are intended for active and intelligent packaging uses, such as inner coatings, wraps, or functional layers, where flexibility, antimicrobial activity, UV protection, and controlled release are prioritized over high load-bearing capacity. Therefore, the mechanical properties achieved in this study are considered adequate for their targeted functionality, while further reinforcement strategies (e.g., multilayer structures or reduced plasticizer content) could be explored in future work to enhance strength if required.

### 3.3 FTIR

**Fig. 3:**
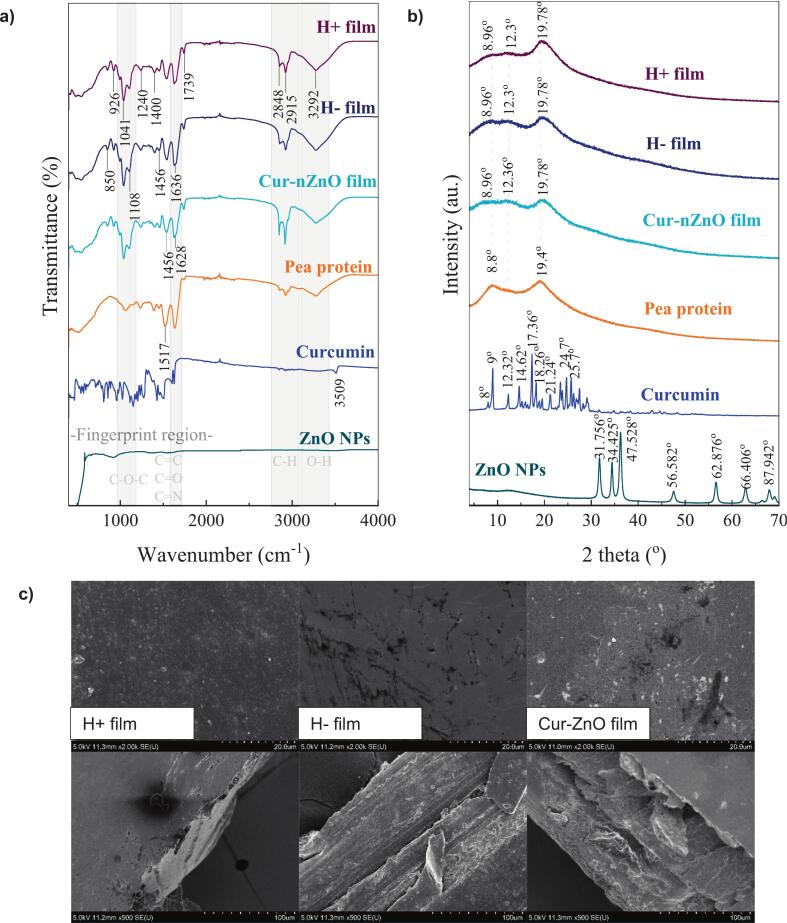
a) FTIR, b) XRD, and c) SEM images of pea protein films. H^+^ film: High-pressure homogenization (HPH) treated, H^−^ film: HPH untreated, Cur-nZnO film: Curcumin and ZnO NPs containing HPH treated.

The IR spectra presented in Fig. 3a provide insights into the chemical structure and molecular interactions within the pea protein-based films and their functional additives, including curcumin and ZnO NPs. All film samples exhibited characteristic protein absorption bands, notably the Amide I (∼1636 cm^−1^) and Amide II (∼1536–1540 cm^−1^) peaks, which correspond to C=O stretching and N—H bending vibrations, respectively. These bands are also present in native pea protein powder, confirming the retention of protein secondary structures in the film network (Melchior et al., 2022). A slight shift and intensity variation in the Amide I–II regions, particularly in Cur–ZnO-loaded films, indicate possible hydrogen bonding and electrostatic interactions between the protein backbone and the functional additives (Huang et al., 2021; Wang et al., 2022). These interactions suggested partial unfolding or reorganization of protein chains, promoting interfacial compatibility. The C—O stretching and C—O—C vibrations observed in the 1041–1080 cm^−1^ range reflect the protein backbone structure and possible interactions with residual polysaccharide-like structures. These peaks appeared sharper and more defined in the HPH^+^ film, indicating improved molecular packing and phase distribution, likely due to the disruptive shear forces of homogenization enhancing dispersion and alignment (Song et al., 2013). Bands at 2925 cm^−1^ and 2848 cm^−1^, assigned to asymmetric and symmetric —CH₂ stretching vibrations, are more intense in Cur–ZnO films, possibly due to the interaction of hydrophobic regions in the protein matrix with the aliphatic domains of curcumin and the surface-bound hydroxyls of ZnO NPs (Aliabbasi et al., 2021). This suggests enhanced hydrophobic domain interactions and surface adsorption effects. A broad and pronounced band around 3292 cm^−1^, corresponding to overlapping O—H and N—H stretching, is especially evident in the HPH^+^ film, pointing to an increase in hydrogen bonding capacity following HPH treatment. This enhanced hydrogen bonding may contribute to greater film compactness and tensile integrity, as corroborated by mechanical property results (Cheng et al., 2022). The Zn—O stretching vibration, typically observed in the 450–500 cm^−1^ range, is weak but present in the Cur–ZnO composite films, confirming the incorporation of ZnO NPs into the matrix (Ran et al., 2022). The reduced intensity of this band may be due to partial embedding or surface shielding by the protein-polysaccharide matrix.

Additionally, curcumin-specific aromatic C=C stretching bands in the 1600–1620 cm^−1^ region appear diminished or shifted in the composite film, suggesting non-covalent interactions such as π–π stacking or hydrogen bonding between Curcumin molecules and aromatic amino acid residues in the protein network (Rachtanapun et al., 2021). The absence of free curcumin peaks also suggests good dispersion or entrapment within the protein phase.

### 3.4 XRD

The XRD patterns (Fig. 3b) reveal the crystalline characteristics and molecular arrangements of pea protein, curcumin, ZnO NPs, and their composite films (H^−^, H^+^, Cur–nZnO). PPI exhibited a broad, low-intensity diffraction peak centered around 2θ ≈ 20°, which indicates a semi-crystalline to predominantly amorphous structure. This pattern is characteristic of globular legume proteins and arises from the coexistence of disordered regions, such as random coils, and loosely packed β-sheet domains (Tang et al., 2023). The same diffuse halo was observed in the H^−^ film, suggesting that film casting without homogenization did not significantly alter the protein's intrinsic molecular organization. In contrast, the H^+^ film displayed a slightly sharper peak ∼20°, indicating a modest increase in short-range molecular ordering. This can be attributed to HPH-induced effects, such as protein unfolding, molecular alignment, and enhanced hydrogen bonding between adjacent chains, which promote tighter packing and localized crystallinity (Chen et al., 2013). In the Cur–nZnO film, the broad amorphous halo typical of pea protein isolate (PPI) is clearly observed, indicating the dominant amorphous nature of the protein matrix. While the sharp crystalline peaks characteristic of ZnO are not as prominent as in the pure ZnO NP pattern, subtle reflections are still present, confirming the partial retention of crystalline ZnO domains within the film.

In contrast, pure curcumin displayed sharp and intense reflections between 2θ ≈ 10–30°, consistent with its highly crystalline nature (Aliabbasi et al., 2021). These peaks were absent in the Cur–nZnO film, implying that curcumin underwent amorphization or molecular dispersion within the protein–nanoparticle matrix. This transformation likely results from non-covalent interactions, such as π–π stacking with aromatic amino acid residues, hydrogen bonding, or encapsulation effects that disrupt the native lattice of curcumin (Rachtanapun et al., 2021; Wang et al., 2022). Similar findings have been reported where curcumin lost its crystallinity upon interaction with biopolymers or nanoparticle surfaces, which can improve its solubility and functional performance in food systems (Li et al., 2023).

### 3.5 SEM

The surface and cross-sectional SEM micrographs (Fig. 3c) reveal distinct morphological differences among the H^+^, H^−^, and Cur–nZnO composite films, which reflect the impact of mechanical processing and nano-bioactive incorporation on film structure.

The H^+^ film surface appeared smoother and more homogeneous than the H^−^ film, suggesting enhanced molecular dispersion and cohesive network formation. This is likely due to HPH, which facilitates protein chain unfolding, promotes intermolecular hydrogen bonding, and reduces phase separation by improving component compatibility. The cross-sectional SEM image further supported this observation by showing a compact, layered internal structure with minimal voids or delaminations, indicative of tighter molecular packing and stronger interfacial adhesion. Similar morphological compaction after HPH has been reported in protein-based edible films, leading to improved tensile strength and barrier properties (Saricaoglu et al., 2018). The Cur–nZnO film surface showed discrete bright spots, attributed to well-dispersed ZnO NPs, confirming their successful incorporation into the film matrix. The cross-sectional image of the Cur–nZnO film exhibited a rougher, more lamellar structure, which may result from the combined effects of ZnO NPs inclusion and curcumin interaction (Huang et al., 2021; Wang et al., 2022). These morphological trends are consistent with XRD and mechanical data, which show that HPH treatment improves structural order and film cohesiveness, while the incorporation of curcumin and ZnO introduces microstructural heterogeneity that balances flexibility, crystallinity, and barrier properties (Rachtanapun et al., 2021; Ran et al., 2022).

### 3.6 Thermal analysis

**Fig. 4:**
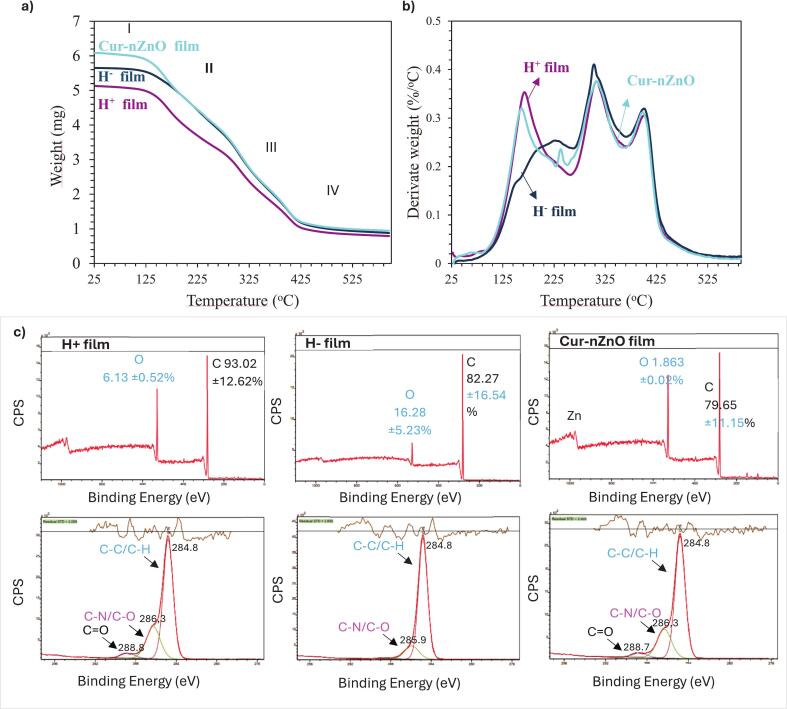
a) Thermal diagram of weight, b) Derivate weight changes, and c) XPS spectra of pea protein films. H^+^ film: High-pressure homogenization (HPH) treated, H^−^ film: HPH untreated, Cur-nZnO film: Curcumin and ZnO NPs containing HPH treated (XPS atomic percentages were obtained from *n* = 3).

The TGA and DTG analyses (Fig. 4a–b) demonstrated that the incorporation of ZnO NPs, curcumin, and HPH treatment markedly influenced the thermal degradation profile of pea protein films. The initial mass loss below 150 °C, related to moisture and volatiles, was slightly lower in Cur–nZnO films, likely due to the water-binding capacity and compact structure induced by ZnO and curcumin, which reduce molecular mobility through hydrogen bonding (Ran et al., 2022; Roy & Rhim, 2020b). In the 150–250 ° C region, degradation involved glycerol volatilization and breakdown of low-molecular-weight compounds. Cur–nZnO films showed improved stability, attributed to enhanced protein–additive interactions such as π–π stacking and hydrogen bonding, limiting volatilization (Akshaykranth et al., 2022). The main degradation (250–400 °C), associated with protein backbone decomposition, shifted slightly to higher temperatures in Cur–nZnO films, indicating a slower degradation process. This is linked to the nanodispersed ZnO acting as a thermal barrier, dissipating heat and delaying structural breakdown (Li, Long, et al., 2024; Li, Wei, et al., 2024). Similar thermal retardation effects of ZnO have been reported in whey, soy, and gelatin-based matrices reinforced with metal oxide NPs (Tang et al., 2019a, 2019b). This behavior contrasts with the findings of Roy and Rhim (2020a), who reported a lower thermal stability in similar biopolymer systems without nanofiller reinforcement. Moreover, HPH treatment further enhanced the thermal resistance, particularly in the H^+^ film. The denser microstructure formed through HPH likely restricted polymer chain motion and enhanced entanglement density and non-covalent cross-linking, thus increasing resistance to thermal scission and decomposition (Saricaoglu et al., 2018). The higher residual mass observed in the Cur–nZnO film at temperatures above 450 °C confirmed the presence of thermally stable inorganic fillers, mainly ZnO, which did not decompose under the applied thermal conditions, as similar to the study of Roy and Rhim (2020a). These residues contribute to the film's thermal resilience and structural integrity, making such systems more suitable for heat-sealing, sterilization, or hot-fill packaging applications.

### 3.7 XPS

XPS was employed to examine the surface elemental composition and chemical bonding states of the film formulations (Fig. 4c). The survey spectra revealed that the H^+^ film had the highest carbon content (93.02 ± 1.52%) and the lowest oxygen (6.13 ± 0.26%) and nitrogen (0.85 ± 0.01%) levels, suggesting a more hydrophobic and compact surface (*p* > 0.0.5). This likely results from enhanced protein alignment and reduced exposure of polar groups due to HPH-induced chain entanglement and phase uniformity (Huang et al., 2021). Conversely, the H^−^ film exhibited lower carbon (82.27 ± 1.02%) and higher oxygen (16.28 ± 0.95%) and nitrogen (1.45 ± 0.05%) contents, indicating a more hydrophilic and disordered surface with exposed polar functionalities due to the absence of mechanical structuring. The Cur–nZnO film showed the highest oxygen (18.63 ± 0.95%) and nitrogen (1.73 ± 0.05%) content, along with a clear Zn signal, confirming successful surface integration of ZnO NPs (*p* > 0.0.5). The elevated oxygen and nitrogen levels reflect the presence of curcumin's polar functional groups, contributing to a more reactive surface (Huang et al., 2021; Li, Long, et al., 2024; Li, Wei, et al., 2024). High-resolution C 1 s spectra displayed a primary peak at 284.8 eV (C—C/C—H) in all samples. H^−^ and Cur–nZnO films also showed stronger peaks at ∼286.3 eV (C—N/C—O) and ∼ 288.7 eV (C=O), indicating increased polar and oxidized carbon species (Zhang, Lan, et al., 2021; Zhang, Li, et al., 2021).

### 3.8 Spectral scan

**Fig. 5:**
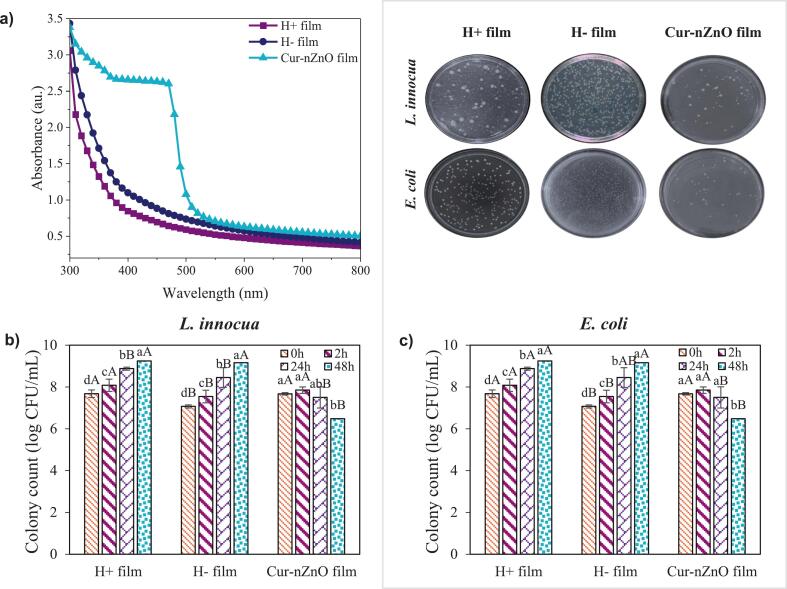
a) Spectral scan, b) Antibacterial activity against *E. coli*, c) Antibacterial activity against *Listeria innocua* of pea protein films. H^+^ film: High-pressure homogenization (HPH) treated, H^−^ film: HPH untreated, Cur-nZnO film: Curcumin and ZnO NPs containing HPH treated (Lowercase letters denote statistically significant differences among time groups, whereas uppercase letters denote statistically significant differences among sample groups. Spectral scan and colony count data were measured in n = 3).

The UV–Vis spectral scan of the films (Fig. 5a) revealed significant differences in optical transmittance depending on both the film composition and the processing method. Among all samples, the Cur–nZnO film exhibited the lowest transmittance across the UV and visible regions (300–600 nm). This behavior confirms the synergistic UV-blocking effects of curcumin and ZnO NPs. Curcumin's polyphenolic chromophore structure absorbs strongly in the UV-A and UV-B regions (λ ≈ 300–450 nm), primarily due to π–π* transitions within its conjugated system (Veerabhadraiah et al., 2022; Zhang, Lan, et al., 2021; Zhang, Li, et al., 2021). ZnO NPs, on the other hand, possess a wide bandgap (∼3.3 eV) and are renowned for their ability to scatter and absorb UV radiation, making them highly effective for enhancing the UV-barrier function of packaging films (Im et al., 2015). The H^+^ film, prepared by HPH, showed lower transmittance than the H^−^ film throughout the 300–600 nm range. This is likely due to HPH-induced matrix densification, which reduces the presence of microvoids and phase separation, resulting in more uniform dispersion of protein chains and potential light-scattering sites.

### 3.9 Water vapor permeability (WVP)

The WVP of pea protein films was significantly influenced by processing and active-agent incorporation. As shown in Table 1, applying HPH reduced WVP, with H^+^ exhibiting a lower WVP than H^−^ (H^−^ = 7.2 ± 0.6 × 10^−10^ g·m / (m^2^·s·Pa), H^+^ = 5.4 ± 0.4 × 10^−10^ g·m / (m^2^·s·Pa)). The lowest WVP was obtained for the Cur-nZnO film (3.9 ± 0.3 × 10^−10^ g·m / (m^2^·s·Pa)), indicating that the combined incorporation of curcumin and ZnO NPs under HPH further improved the water vapor barrier.

**Table 1:** Water vapor permeability (WVP) of pea protein films as affected by HPH processing and Cur–nZnO incorporation. H^+^ film: High-pressure homogenization (HPH) treated, H^−^ film: HPH untreated, Cur-nZnO film: Curcumin and ZnO NPs containing HPH treated. (Lowercase letters denote statistically significant differences among sample groups).

Film samples	WVP (g·m / (m^2^·s·Pa))
H^−^ film	7.2 ± 0.6 × 10^−10 a^
H^+^ film	5.4 ± 0.4 × 10^−10 b^
Cur-nZnO film	3.9 ± 0.3 × 10^−10 c^

The decrease in WVP observed for H^+^ relative to H^−^ can be attributed to HPH-induced protein structuring, which is expected to enhance dispersion of protein aggregates and promote a more homogeneous and compact network (Guo et al., 2023). A denser microstructure typically reduces the availability of continuous pathways for water vapor diffusion and increases the effective tortuosity, thereby lowering permeability. This interpretation is consistent with the microstructural evidence (SEM) suggesting improved matrix uniformity after HPH treatment.

The additional reduction in WVP for Cur-nZnO suggests a synergistic barrier effect arising from the presence of ZnO NPs and curcumin within the HPH-structured protein matrix. Well-dispersed inorganic nanoparticles commonly decrease water vapor transport by introducing an impermeable phase and forcing diffusing molecules to follow a longer, more tortuous route (Huang et al., 2021). In parallel, the relatively hydrophobic nature of curcumin may decrease the water affinity of the matrix and limit moisture sorption, which is a key driver of water vapor transmission in hydrophilic biopolymer films Roy and Rhim (2020a). Moreover, under HPH, improved dispersion of both curcumin and ZnO is likely to minimize agglomeration-induced defects, supporting the formation of a tighter interfacial region that further restricts mass transfer. Overall, these results indicate that processing (HPH) and dual-agent incorporation (Cur and nZnO) can be leveraged to tune the WVP of pea protein films through microstructure control.

### 3.10 Antibacterial activity

The antibacterial performance of the films against *L. innocua* and *E. coli* was evaluated over a 48-h incubation period (Fig. 5b–c). Among all samples, the Cur–nZnO film exhibited the most pronounced antimicrobial effect, reducing *L. innocua* and *E. coli* populations by approximately 1.3 and 1.6 log CFU/mL, respectively, compared to the initial inoculum. This significant inhibition is attributed to the synergistic antimicrobial mechanisms of curcumin and ZnO NPs.

ZnO NPs exert their bactericidal effect through multiple pathways: i) Release of Zn^2+^ ions, which disrupt enzymatic and metabolic processes; ii) Generation of reactive oxygen species (ROS) via photocatalytic activity, leading to oxidative damage of lipids, proteins, and DNA; iii) Direct membrane disruption, due to nanoparticle–cell surface interactions (Huang et al., 2021). Curcumin, as a natural polyphenol, contributes to antimicrobial action by: i) inhibiting bacterial membrane proteins and ATPase activity; ii) causing protein denaturation and membrane depolarization; iii) interfering with quorum sensing and biofilm formation in both Gram-positive and Gram-negative bacteria (Akshaykranth et al., 2023). In contrast, both H^+^ and H^−^ control films, which lack inherent antimicrobial agents, allowed substantial bacterial growth over the 48-h period. These findings clearly demonstrate that protein-based films alone lack sufficient antimicrobial capacity unless bioactive agents are integrated.

### 3.11 Release of Curcumin and ZnO NPs from films into food simulants

**Fig. 6:**
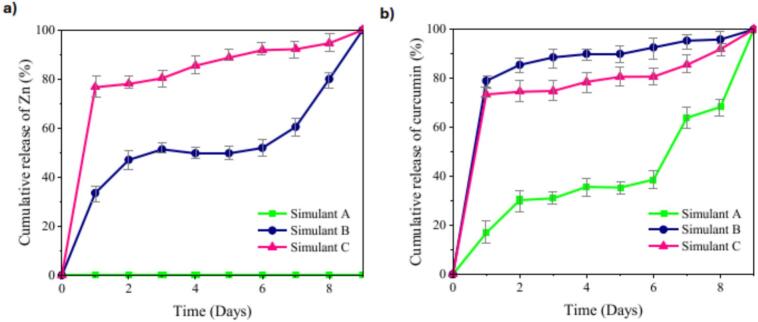
Cumulative relase of a) curcumin and b) ZnO in Cur-nZnO film under simulated conditions, Simulant A: Oleic acid as a vegetable oil, Simulant B: Ethanol, 10% (*v*/v), Simulant C: Acetic acid, 0.5 N. H^+^ film: High-pressure homogenization (HPH) treated, H^−^ film: HPH untreated, Cur-nZnO film: Curcumin and ZnO NPs containing HPH treated [Release experiments were conducted in n = 3].

The release behavior of curcumin and ZnO NPs from the Cur–nZnO composite films was evaluated in three standard food simulants—10% ethanol (Simulant A, aqueous), 3% acetic acid (Simulant B, acidic), and oleic acid (Simulant C, fatty)—to simulate diverse food contact environments (Figs. 6a–b). This approach aimed to assess how different physicochemical conditions affect the diffusion kinetics and matrix interactions of the active agents.

The release of curcumin followed distinct profiles depending on the simulant, as described by the Peleg model, which captures initial release rate (*k₁*) and maximum release extent (*k₂*). In simulant C, curcumin showed the fastest initial release (*k₁* = 3.38), but a moderate equilibrium release (*k₂* = 0.67). This indicates rapid diffusion into the lipid phase, but partial retention within the protein matrix—likely due to hydrophobic interactions and entrapment in dense film regions (Rachtanapun et al., 2021). Lipid environments tend to extract non-polar compounds more readily, although curcumin's crystalline tendency may limit its total migration (Wang et al., 2022). In simulant A, curcumin exhibited the slowest release (*k₁* = 0.16; *k₂* = 0.56), likely due to its limited solubility in hydroalcoholic media and intermolecular stabilization via hydrogen bonding or π–π stacking with the protein network (Dong et al., 2023; Madian et al., 2023). In contrast, simulant B provided the most favorable release conditions (*k₁* = 0.63; *k₂* = 1.11), possibly due to: i) Swelling of the protein matrix, ii) Partial electrostatic disruption of protein–curcumin interactions, and iii) Enhanced film porosity or erosion under acidic conditions (Karabulut & Feng, 2024).

The Zn release from the Cur–nZnO films was evaluated in simulant A and B using zero-order and first-order kinetic models. In simulant A, Zn release followed a near-linear profile, fitting best with the zero-order model (*R*^*2*^ = 0.814), suggesting a steady and sustained release, typical of dense matrices where diffusion is the rate-limiting step. In simulant B, however, both models exhibited poor correlation (*R*^*2*^ = 0.526 and 0.462, respectively), indicating a more complex release mechanism-possibly involving: i) Film swelling and partial hydrolysis, ii) Increased ionic mobility and Zn^2+^ solubilization, or iii) Acid-induced matrix erosion (El Fawal et al., 2020; S. Tang et al., 2019b). To better understand the migration process, Fick's second law was applied assuming a slab model with 0.20 mm thickness, yielding diffusion coefficients (*D*) of 1.51 × 10^−9^ m^2^/s in Simulant A, and 6.21 × 10^−9^ m^2^/s in Simulant B. These values suggest faster Zn migration in acidic conditions, which is consistent with previous reports indicating that acid exposure can disrupt matrix integrity, increase matrix hydration, and facilitate ion exchange processes.

These findings reinforce that food matrix composition and pH play a crucial role in controlling the release kinetics of active agents. The Cur–nZnO film's tunable migration behavior positions it as a promising candidate for pH-sensitive, controlled-release packaging applications, especially in high-moisture or acidic foods. Although water vapor permeability, oxygen permeability, and water solubility are critical parameters for conventional packaging performance, the present study primarily focused on elucidating the structure–function–release relationships of high-pressure-homogenized pea protein films incorporating ZnO nanoparticles and curcumin.

It should be noted that the present release study was designed to provide a mechanistic evaluation of curcumin and zinc migration using standardized food simulants, rather than to assess food-specific sensory or toxicological outcomes. The impact of curcumin release on food flavor is highly dependent on the food matrix, concentration threshold, and formulation, and therefore requires dedicated sensory evaluation in real food systems. Although a full toxicological/migration assessment is beyond the scope of this work, the Zn^2+^ release profiles are briefly contextualized for practical relevance. The cumulative release is presented as % of the total Zn incorporated, and thus the absolute Zn^2+^ amount potentially available depends on the film mass/area and the specific contact/consumption scenario. Given the ZnO loadings used here (0.075–0.375% *w*/w relative to total solids), a conservative worst-case estimate can be obtained by multiplying the total Zn content in the film by the measured cumulative release fraction. This qualitative benchmarking is provided to aid interpretation and should not be taken as a definitive safety conclusion. For adults, tolerable upper intake levels (ULs) for zinc are frequently reported as 40 mg/day (US National Academies/NIH ODS) and 25 mg/day (Panel & Nda, 2014).

In controlled-release packaging systems, various encapsulation and dispersion strategies, such as emulsion-based systems, nanoliposomes, polymeric microcapsules, and metal–organic frameworks (MOFs), have been widely explored to enhance the stability and release control of functional compounds (Li et al., 2023; Li, Long, et al., 2024; Li, Wei, et al., 2024; McClements, 2018). These approaches generally rely on carrier-based architectures, in which bioactives are associated with an additional delivery vehicle. Previous studies have reported multifunctional packaging materials based on different polymer matrices and processing strategies, including chitosan films reinforced with TiO₂ nanoparticles and red apple pomace extract, as well as PLA/ZnO composite membranes produced via ultrasonication and electrospinning (Lan et al., 2021; Zhang, Lan, et al., 2021; Zhang, Li, et al., 2021). These approaches mainly rely on rigid matrices or fibrous architectures to enhance mechanical and barrier properties.

In contrast, the present study employs a high-pressure homogenization–assisted, matrix-integrated approach in an edible pea protein film system, enabling simultaneous protein network restructuring and active compound dispersion. This strategy facilitates controlled release within a flexible, biodegradable, and food-compatible film matrix, offering a complementary design route for active packaging applications.

## 4 Conclusion

This study demonstrated the successful development of multifunctional edible films incorporating high-pressure homogenized pea protein, curcumin, and ZnO NPs. High-pressure homogenization significantly enhanced the film matrix by promoting protein chain alignment and improving network homogeneity and partial crystallinity. Structural characterization suggested the effective molecular dispersion of curcumin and the stable crystalline incorporation of ZnO within the biopolymer matrix. In addition, water vapor permeability (WVP) was reduced by HPH processing and further decreased upon Cur–nZnO incorporation, indicating an improved moisture barrier performance of the films. The resulting films exhibited promising antimicrobial properties and stimulus-responsive release behavior, varying according to the type of food simulant. Notably, curcumin showed enhanced release in acidic conditions, while zinc exhibited diffusion-controlled migration with higher mobility in acetic acid. These functionalities highlight the potential of Cur–nZnO biocomposite films as active packaging materials capable of delivering antimicrobial protection alongside improved structural performance. To support safe translation toward food-contact applications, future studies should systematically evaluate the potential toxicological effects of ZnO nanoparticles and confirm the absence of cytotoxicity at the applied concentrations using appropriate in vitro and/or in vivo models. Future research should also focus on evaluating the gastrointestinal stability of released bioactives, assessing biodegradability under real-world storage and disposal conditions, and optimizing mechanical and sensory attributes for commercial use.

## CRediT authorship contribution statement

**Gulsah Karabulut:** Writing – original draft, Visualization, Validation, Project administration, Methodology, Investigation, Formal analysis, Conceptualization. **Vedant Mundada:** Methodology, Investigation, Formal analysis. **Ragya Kapoor:** Methodology, Investigation, Formal analysis. **Hao Feng:** Writing – review & editing, Supervision, Resources, Project administration.

## Declaration of competing interest

The authors declare that they have no known competing financial interests or personal relationships that could have appeared to influence the work reported in this paper.

## Data Availability

Data will be made available on request.
